# Potential Immunohistochemical Biomarkers for Grading Oral Dysplasia: A Literature Review

**DOI:** 10.3390/biomedicines12030577

**Published:** 2024-03-05

**Authors:** Jakub Zdrojewski, Monika Nowak, Kacper Nijakowski, Jakub Jankowski, Andrea Scribante, Simone Gallo, Maurizio Pascadopoli, Anna Surdacka

**Affiliations:** 1 Department of Conservative Dentistry and Endodontics, Poznan University of Medical Sciences, 60-812 Poznan, Poland; jzdrojewski@ump.edu.pl (J.Z.); mnowak@ump.edu.pl (M.N.); annasurd@ump.edu.pl (A.S.); 2 Student’s Scientific Group, Department of Conservative Dentistry and Endodontics, Poznan University of Medical Sciences, 60-812 Poznan, Poland; jjankowski41@wp.pl; 3 Unit of Orthodontics and Pediatric Dentistry, Section of Dentistry, Department of Clinical, Surgical, Diagnostic and Pediatric Sciences, University of Pavia, 27100 Pavia, Italy; simone.gallo02@universitadipavia.it (S.G.);; 4 Unit of Dental Hygiene, Section of Dentistry, Department of Clinical, Surgical, Diagnostic and Pediatric Sciences, University of Pavia, 27100 Pavia, Italy

**Keywords:** oral dysplasia, oral epithelial dysplasia, immunohistochemistry, histological grading, immunoexpression

## Abstract

Oral cancer is a prevalent global health issue, with significant morbidity and mortality rates. Despite available preventive measures, it remains one of the most common cancers, emphasising the need for improved diagnostic and prognostic tools. This review focuses on oral potentially malignant disorders (OPMDs), precursors to oral cancer, specifically emphasising oral epithelial dysplasia (OED). The World Health Organisation (WHO) provides a three-tier grading system for OED, and recent updates have expanded the criteria to enhance diagnostic precision. In the prognostic evaluation of OED, histological grading is presently regarded as the gold standard; however, its subjectivity and unreliability in anticipating malignant transformation or recurrence pose notable limitations. The primary objective is to investigate whether specific immunohistochemical biomarkers can enhance OED grading assessment according to the WHO classification. Biomarkers exhibit significant potential for comprehensive cancer risk evaluation, early detection, diagnosis, prognosis, and treatment optimisation. Technological advancements, including sequencing and nanotechnology, have expanded detection capabilities. Some analysed biomarkers are most frequently chosen, such as p53, Ki-67, cadherins/catenins, and other proteins used to differentiate OED grades. However, further research is needed to confirm these findings and discover new potential biomarkers for precise dysplasia grading and minimally invasive assessment of the risk of malignant transformation.

## 1. Introduction

Oral cancer is becoming more and more frequent worldwide [1]. Despite the widely available prevention, it is one of the most common cancers in the world, with 476,125 new cases and 225,900 deaths in 2020 [2]. Among the causes of carcinogenesis in the oral cavity, tobacco smoking or chewing, alcohol consumption, occupational exposure, risky sexual behaviour, genetic factors, and environmental pollution are widely mentioned [3]. Smoking is the most prominent risk factor for oral cancer due to the carcinogenic chemicals in cigarette smoke, including nitrosamines, benzopyrenes, and aromatic amines [4]. The risk of oral cancer is three times higher in smokers compared to non-smokers. In addition, the combination of cigarette smoking and frequent heavy alcohol consumption increases the risk of developing cancer by several times [5,6].

Neoplastic lesions are often preceded by oral potentially malignant disorders (OPMDs) [7]. The World Health Organisation’s (WHO) classification of head and neck cancers defines OPMDs as “clinical symptoms carrying the risk of developing oral cancer, whether clinically definable precursor lesions or clinically normal mucosa” [8]. This group includes lesions such as leukoplakia, oral lichen planus, and oral lichenoid lesions [9]. Until recently, oral epithelial dysplasia (OED), proliferative verrucous leukoplakia, submucous fibrosis, and HPV-associated dysplasia were classified as OPMDs [10]. The histological presence of OED is currently the strongest predictor of malignant transformation in OPMDs [11]. According to the WHO classification, OED is characterised as “a spectrum of architectural and cytological epithelial changes resulting from the accumulation of genetic alterations, usually arising in a range of OPMD and indicating a risk of malignant transformation to OSCC” [9]. These structural changes reflect the loss of normal maturation and stratified epithelium [12].

Therefore, a biopsy is conventionally performed to assess precancerous changes (dysplasia) in the tissue and obtain a histopathological diagnosis of a potentially malignant disease. The terminology of dysplasia was re-adopted by the WHO in the Classification of Tumours of the Oral Cavity and Oropharynx in 2005. However, instead of using the term dysplasia, some authors suggest employing the term squamous intraepithelial neoplasia (SIN) or variations such as oral intraepithelial neoplasia (OIN) [13], which are modifications of cervical pre-malignant lesions [14]. This change in terminology to OIN aims to avoid confusion with the WHO’s term of CIS (carcinoma in situ) and to emphasise the characteristics of OSCC that differ from those of SCC of the uterine cervix [15]. The WHO refrained from endorsing this suggestion. The decision against adopting the SIN terminology stemmed from its perceived inadequacy in clarifying the situation in a manner significant enough to replace the globally utilised concept of dysplasia [16]. Furthermore, it was not demonstrated at that time that many OPMDs lead to cancer [17,18].

To assess the extent of dysplasia, a set of grading criteria was implemented to categorise the progression of the lesion. According to the WHO three-tier OED classification, dysplasia is classified as mild, moderate, or severe, considering both architectural features (tissue changes) and cytological alterations (changes in individual cells/cytological pattern) [14]. In the most recent WHO classification as of 2022, the OED grading criteria were expanded to encompass additional architectural and cytologic features, as detailed in Table 1 [19]. This expansion aims to enhance the diagnostic precision of dysplasia, emphasising that architectural features alone may indicate the presence of dysplasia. Despite the inherent challenges in dysplasia grading, the WHO maintains a three-tiered grading system [10,20].

Moreover, the binary classification system would be an alternative approach to the WHO classification. This system categorises OED into low- and high-risk dysplasia, utilising a quantitative threshold of dysplastic pathological features and aiming to enhance reliability [19]. Furthermore, the binary system offers promising results in predicting malignant transformations, overcoming “opt-out” judgments associated with the four-scale or five-scale grading system [21,22]. While this may facilitate disease categorisation and reduce observer variability, the clinical prognostic value remains largely untested and widespread acceptance of this system necessitates additional international validation before it can be fully endorsed [9,21,22,23].

In OED prognostication, histological grading is found as the current gold standard but is subjective and unreliable to predict malignant transformation or recurrence [24]. Therefore, we aimed to answer whether alterations in the expression of specific immunohistochemical biomarkers could help to facilitate OED grading assessment according to the WHO classification. For this purpose, we prepared a literature review covering original articles published between 2017 and 2022 and indexed in databases, such as PubMed, Scopus, and the Web of Science.

## 2. Discussion of Potential Immunohistochemical Biomarkers in Grading of Oral Epithelial Dysplasia

Researchers are still conducting studies that will establish a biomarker that can unambiguously diagnose and differentiate between the stages of dysplasia. Biomarkers can be genes, proteins, or other substances whose levels or presence are tested to detect cell changes [25]. Not all cells affected by carcinogenesis are the same, as they may present gene changes or differences in the levels of given metabolites and proteins [26]. Detection technologies have developed significantly in recent decades, including sequencing, nanotechnology, or methods determining circulating tumour DNA/RNA or exosomes [27]. The clinical applications of biomarkers are broad. They can be used as tools for cancer risk assessment, screening and early cancer detection, accurate diagnosis, prognosing patients’ condition, and predicting responses to treatment [28]. Also, they help in the optimisation of the treatment process. This is essential for targeted therapy, as it is only effective in patients with specific cancer genetic mutations, and biomarkers are used to identify these subgroups [29]. Further research is required to overcome the scientific challenges of developing new biomarkers with greater sensitivity, specificity, and positive predictive value.

Interestingly, many biomarkers are emerging in studies regarding the differentiation of dysplasia grades. De Vicente et al. [30] observed an association between NANOG (a key regulator of pluripotency and self-renewal in embryonic and adult stem cells) and the grade of dysplasia. It was noted that expression of NANOG increased with the grade of dysplasia. The importance of NANOG was also confirmed in the study by Grubelnik et al. [31], which stated that this marker can be used to differentiate dysplasia grades. NANOG protein detection has a diagnostic potential for oral high-grade dysplasia, distinguishing it from low-grade dysplasia and non-neoplastic reactive lesions.

The study by Wang et al. [32] showed that significantly increased Orai1 and STIM1 protein levels were noted in OPMD with mild, moderate, and severe OED in comparison with normal oral mucosa. Orai1 is calcium release-activated calcium modulator 1. This protein is a membrane calcium channel subunit activated by the calcium sensor STIM1 when calcium stores are depleted. Disruption of normal intracellular Ca^2+^ is reported to be associated with the formation of cancer in some studies [33].

Given the different mechanisms of dysplasia development, the sophistication of the malignant transformation processes in the cells, and the individual changes in each person subjected to different environmental factors, it is very difficult to isolate a single comprehensive biomarker. For this literature review, the most commonly mentioned biomarkers were proteins, such as p53, Ki-67, cadherins/catenins, and others.

### 2.1. Biomarkers Related to Cell Division and Proliferation

The cell cycle is regulated by the activity of various cyclins and cyclin-dependent kinases (Cdks). Cyclins form a complex with Cdks, and complex formation results in the activation of the Cdk active site. Cyclins without Cdk activation have no enzymatic activity but have binding sites for some substrates [34]. Cyclins are some of the most important cell cycle regulatory proteins and are linked to a specific phase of the cycle [35]. Both cyclins and their associated proteins are currently the subject of intense research, as perturbations of their expression and regulation can lead to tumorigenesis [36]. The majority of findings have reported on the overexpression of cyclins D and E in the development of many types of cancer [37].

In many studies, p63 and CD31 are the primarily examined markers. The p63 protein in normal cells is found in the basal layer of squamous epithelium [38]. Bavle et al. [39] found that p63 expression rose with increased severity of dysplasia and increased expression in suprabasal cells. The studies showed that p63 is required to maintain cell proliferation. It was observed that as the severity of dysplasia rose, the proliferation rate increased; however, cell differentiation was jeopardised [40]. As the disease progressed, the number of blood vessels increased and angiogenesis occurred. This is one of the factors that plays an important role in tumour growth and metastasis, providing nutrition to the developing tumour [41]. CD31 protein is a marker of angiogenesis, so it was used to detect vascular changes near the epithelium. The correlation of p63 with CD31 added value to the categorisation of leukoplakic lesions in the cases of low and moderate dysplasia [39].

Patel et al. [42] assessed p63 expression in different grades of dysplasia and Cyclin D1 expression. Cyclin D1 is classified as a proto-oncogene. *P63* expression showed no statistically significant differences in different grades of dysplasia, and cyclin D1 showed only statistically significant differences between severe and mild grades of dysplasia. Gupta et al. [43] used VEGF and CD34 as dysplasia markers. The study evaluated the percentage of VEGF immunoreactivity, the intensity of VEGF staining, and CD34 immunostaining. The expression of VEGF and CD34 increased significantly during the transition from normal oral mucosa to severe OED.

CD44—cluster of differentiation 44—is a transmembrane glycoprotein [44]. Venkat Naga et al. [45] used a cluster of differentiation 44 (CD44) antibody to assess the correlation between this marker and oral dysplasia grading. The authors compared four groups: control tissue, mild epithelial dysplasia, moderate epithelial dysplasia, and severe epithelial dysplasia. A comparison of the groups showed statistically significant results. It suggested that CD44 may be a useful marker for diagnosing dysplastic lesions.

Interestingly, Aravind et al. [46] evaluated the osteopontin (OPN) expression in premalignant and malignant lesions. The authors observed a progressive increase in OPN expression, which was seen with increasing grades of dysplasia. Osteopontin seemed to be a promising biomarker in predicting the malignant potential of a premalignant lesion. Osteopontin, a phosphorylated sialoprotein, is a component of the mineralised extracellular matrices of bones and teeth [47] that has many functions in inflammation, immune responses, wound healing, cell adhesion, and cell migration through interactions with integrins and CD44 variants [48].

**Figure 1:**
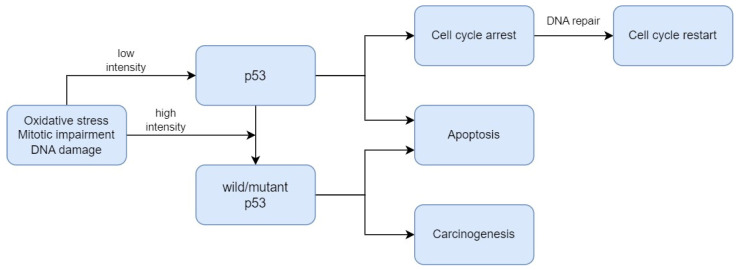
The mechanism of p53 regulation in DNA damage response.

*P53*, also known as *TP53*, is a gene that encodes a protein that regulates the cell cycle and, therefore, acts as a tumour suppressor, regulating cell division by stopping cells from growing and proliferating too rapidly or in an uncontrolled manner [49]. As presented in Figure 1, p53 plays a critical role in the regulation of the DNA damage response. Under normal conditions, p53 is expressed at an extremely low level. The regulation of p53 activity is caused by the MDM2 protein, which contributes to the proteasomal degradation of this suppressor [50]. When DNA damage or energetic stress occurs in a cell, p53 expression is induced, causing the cell cycle to stop. This is a chance for the repairment processes, or the cells will develop apoptosis. The most important purpose of this protein is to eliminate cancer-prone cells from the replication pool [51]. When DNA damage, mitotic impairment, and oxidative stress are excessive, the p53 protein can be mutated to wild-type p53 protein (wtp53), which is inactivated under physiological conditions [52]. Mutations in the *P53* gene and the functions of wtp53 expression have been linked to various human cancers [49].

Researchers demonstrated p53’s role in differentiating grades of dysplasia. Pandya et al. [53] showed that the difference in expression was statistically significant between mild and severe dysplasia. The difference in TP53 expression between mild and severe dysplasia was statistically significant, according to Patil et al. [54]. The expression also increased with the increasing grades of epithelial dysplasia. Deregulation of this oncosuppressive protein may be important for the liability of the lesions to carcinogenesis. In the study by Sawada et al. [55], the higher the grade of dysplasia, the more frequently a TP53 mutation was observed. Imaizumi et al. [56] assessed p53 expression by immunofluorescence as a biomarker to differentiate between oral squamous epithelial lesions. The study consisted of 129 archival oral biopsy samples, including 18 benign squamous lesions, 37 low-grade dysplasias, 22 high-grade dysplasias, and 52 OSCCs. The authors found that the expression of p53 can be a valuable biomarker that helps to estimate the grade of oral epithelial dysplasia.

ΔNp63 is in the p53 family and is a p63 isoform, guiding the maturation of these stem cells through the regulation of their self-renewal and terminal differentiation. Yes-associated protein (YAP) is an oncoprotein in the cytoplasm in an inactive form [57]. YAP moves to the cell nucleus and activates the transcription of genes responsible for cell division and apoptosis [58]. Ono et al. [59] assessed the correlation between the expression of ΔNp63 and YAP and the grade of oral dysplasia. The authors found that in oral dysplasia, the expression of YAP and ΔNp63 was higher in high-grade than in low-grade disease. YAP and ΔNp63 expression correlated with grades of oral dysplasia.

The Ki-67 protein is widely used as a marker of human cancer cell proliferation [60]. Ki-67 plays a role in interphase and mitotic cells, and its distribution changes during the cell cycle. These localisations are associated with distinct functions [61]. Increased tumour cell proliferation is considered a significant natural factor in cancer detection. Ki-67 plays a significant role in cancer formation due to its positive association with tumour proliferation and invasion [62]. Ki-67 is the most suitable biological marker of mitotic activity due to its expression in the nucleus in a specific cell cycle period [63].

Mutations of P53 and high levels of Ki-67 protein are frequently observed in various types of human cancer. Ki-67 shows a stronger association with poor tumour differentiation and negatively affects patients’ survival in advanced stages [64]. Both P53 mutational status/type and high Ki-67 can also significantly impact overall survival [65]. The expression of p53 and Ki-67 increases as normal oral mucosa becomes dysplastic and undergoes malignant transformation [66]. Co-expression of p53 and Ki-67 is related to larger tumours and metastasis to lymph nodes; thus, this observation suggests that it can be used to identify high-risk lesions [67].

In their study, Kamala et al. [68] observed an increase in Ki-67 expression with the severity of dysplasia. The Ki-67 antigen can be used as a marker for histological evaluations of OED. According to Dash et al. [69], as the severity of OED increases, the number of cells showing positive Ki-67 expression also increases. This is also confirmed by Mondal et al. [70], who found that the differences in Ki-67 expression were statistically significant between normal mucosa and mild dysplasia, as well as between mild, moderate, and severe dysplasia. Ki-67 not only detects the hyperactive cells in OED, but its expression of Ki-67 can also be comparable to the clinical course or prognostication of a disease.

According to the study by Takkem et al. [71], Ki-67 expression was restricted to the basal layers of normal oral epithelium, while Ki-67-positive cells in OED were localised in the basal, suprabasal, and squamous layers; Ki-67 expression was increased in patients at high case risk. Ki-67-positive cells in well-differentiated OSCC were mainly located at the periphery of tumour nests; in moderately differentiated OSCC, they were located both at the periphery and in part of the centre of tumour nests, while they were scattered in the most poorly differentiated lesions. The study by Kamala et al. [68] aimed to determine the degree and pattern of expression of aberrant Ki67 in OSMF. The study confirmed a statistically significant correlation between the expression of Ki-67 with the clinical and histological grading of OSMF and the histological grading of OSCC.

Moreover, Swain et al. [72] examined Ki-67 with MCM2 expression in OED, OSCC, and normal mucosa. The study confirmed that the expression of these proteins increased progressively. The expression profile of MCM 2 and Ki-67 was increased with the increasing grades of epithelial dysplasia. In their studies, Gadbail et al. [73,74] used Ki-67, CD105, and α-SMA antigen to differentiate the OED grades. The expressions of Ki-67, CD105, and α-SMA markers complement the binary grading system of OED. Ki-67 showed significant increases from normal oral mucosa to low-grade and high-grade epithelial dysplasia.

Additionally, Suwasini et al. [75] found a statistically significant association between p53 and Ki-67. The results highlighted the potential use of the p53 protein and the Ki-67 antigen as significant molecular markers for early PMD detection and OSCC risk. This observation was also confirmed by Leung et al. [76]—Ki-67 and p53 were significantly increased with higher histological grades of OD. These observations showed the role of DNA-replicative stress in higher grades of dysplasia and transformation from OD to OSCC.

Monteiro et al. [77] analysed the immunoexpression of BubR1, Mad2, Bub3, Spindly, and Ki-67 proteins in 64 oral biopsies. Spindly is a protein that targets dynein/dynactin to kinetochores in mitosis. The authors observed that the expression of Spindly was significantly correlated with a high Ki-67 score and the grade of dysplasia. This observation confirmed that the expression of Ki-67 protein is associated with an increased risk for malignant transformation.

Stathmin is a member of a family of proteins that plays important roles in regulating the microtubule cytoskeleton [78]. This protein regulates microtubule dynamics by promoting the depolymerisation of microtubules and/or preventing the polymerisation of tubulin heterodimers [79]. Vadla et al. [80] evaluated the role of stathmin in OSCC and oral dysplasia and the correlation of stathmin expression with dysplasia grading. The study presented a statistically significant correlation between increased grades of oral dysplasia and expression levels of stathmin. This study confirmed the positive role of stathmin in disease progression and suggested that stathmin could be an early diagnostic biomarker for oral dysplasia.

### 2.2. Biomarkers Related to Epithelial–Mesenchymal Transition (EMT)

Epithelial stem cells maintain tissues throughout adult life and are controlled by epithelial–mesenchymal interactions to balance cell production and loss. A defining characteristic of an epithelium is the close contact that these cells have with the underlying mesenchyme [81]. Polarised epithelial cells normally interact with the basement membrane, causing several biochemical changes that enable them to adopt a mesenchymal cell phenotype, including enhanced migratory capacity, invasiveness, elevated resistance to apoptosis, and greatly increased production of ECM components. This biological process is called an epithelial–mesenchymal transition (EMT) [82]. This transformation can occur in physiological processes during embryogenesis, organ development, and tissue regeneration, as well as in tumorigenesis and cancer progression, including tumour cell invasion and metastasis [83].

Mesenchymal stem cells are stromal cells capable of self-renewal and multilineage differentiation. They show a greater ability to infiltrate the capillaries at the site of the primary tumour lesions [84,85]. This mechanism is a critical mechanism for the acquisition of the malignant phenotype in neoplastic epithelial processes. This subtype accompanies the formation of distant metastases, where, in secondary foci, cells change their phenotype through a reverse mesenchymal–epithelial transition (MET) [86,87].

The role of EMT in OSCC is to transform normal epithelial cells into malignant mesenchymal cells by losing intercellular adhesion, causing metastatic progression and infiltration [88]. In the epithelial stage, tumour cells are cubic and adherent to each other. Also, in this stage, tumour cells show positive E-cadherin expression and negative vimentin expression. In the mesenchymal stage, the tumour cells show higher vimentin expression, but the expression of E-cadherin is repressed. The tumour cells are fibroblast-like and lose their cell–cell junctions [89].

The hallmark of EMT is the upregulation of N-cadherin followed by the downregulation of E-cadherin, and this process is regulated by a complex network of signalling pathways and transcription factors. The breakdown of cell–cell connections is caused by a change in cadherin expression (E-cadherin replaced by N-cadherin). Then, cells lose their apical–basal polarity, which is converted into a front–rear polarity. The downregulation of E-cadherin is often found in malignant epithelial cancers. N-cadherin indicates ongoing EMT and its expression has been correlated with the development of various types of carcinoma [90].

Also, MMPs can induce EMT, contributing directly to cell migration and invasion by degrading specific substrates and implicating many steps of carcinogenesis, including primary tumour growth, angiogenesis, basal membrane and stroma invasion, and metastatic progression [91]. Structural and functional support to the cell is provided by vimentin-filamentous protein. In the early stages of cancer, vimentin is at a very low level. Its concentration increases when the tumour invades the surrounding areas [92].

**Figure 2:**
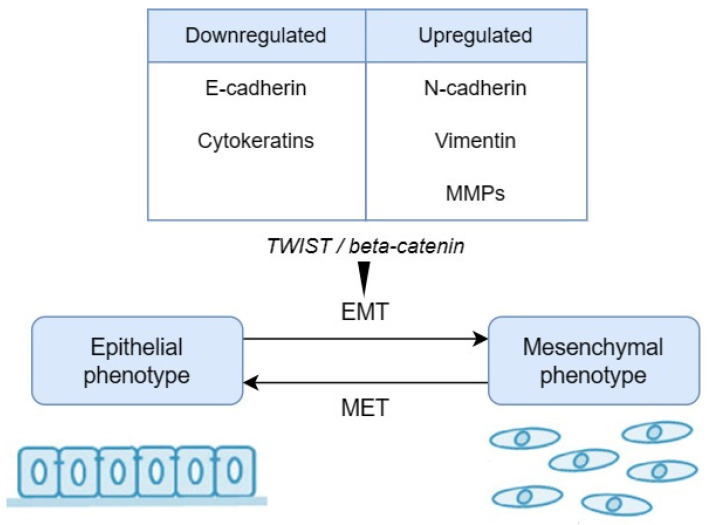
The process of epithelial–mesenchymal transition (EMT).

Remodelling the cytoskeleton results in altered cell morphology and increased motility. EMT is dictated by a series of changes in the expression levels of proteins regulated by the activity of proteins responsible for intercellular interactions (Figure 2). Markers of EMT are proteins specific to the epithelial phenotype, e.g., E-cadherin, mucin-1, cytokeratins, occludin, or desmoplakin, whose activity is reduced. As a result of EMT, the levels of N-cadherin, vimentin, fibronectin, or vitronectin are increased [93]. The expression of the EMT-associated protein markers can be used by pathologists as specific indicators of risk of malignancy processes. Moreover, the ability to adapt to different environmental conditions or in the presence of chemotherapeutics is the main characteristic of malignant tumours and is closely linked to EMT. This relationship can be helpful for oncological therapeutic strategies [94]. Understanding EMT and MET may help to identify specific markers to distinguish normal stem cells from cancer stem cells in the future [86].

One of these biomarkers are cytokeratins (CKs). CKs are keratin proteins located in the intracytoplasmic cytoskeleton of epithelial tissue. They are an important component of the intermediate filaments that help cells resist mechanical loads [95]. Batool et al. [96] found a strong correlation between the intensity of CK5\6 staining and the different stages of dysplasia. Additionally, this marker allows for the differentiation of healthy mucosa from dysplastic mucosa. A gradual increase in staining intensity for CK5\6 was observed with increasing grades of dysplasia. They found a highly significant association with CK5\6 immunopositivity and transforming normal mucosa into various grades of oral dysplastic lesions. Also, CK19 belongs to a family of keratins. CK19 is an odontogenic epithelial marker reported to exhibit increased expression in various cancers, including OSCC [97]. Rajeswari et al. [98] noticed an increased expression of CK19 in severe dysplasia, but in mild and moderate dysplasia, CK19 expression was lower than the normal mucosa. This study showed that CK19 cannot be a marker to assess the grading of dysplasia.

β-Catenin regulates cell adhesion and migration as an intercellular junction-forming element in complex with E-cadherin [99]. Intercellular junctions determine their polarity and enable tissue integrity, growth, and maturation [100]. They enable interaction and signal transmission between neighbouring cells and between neighbouring cells and the extracellular matrix. Weakening intracellular junctions can lead to the disruption of cell cycle control, resulting in the separation of individual cells from the primary tumour, thus creating the conditions for tumour metastasis [101]. E-cadherin is produced on the surface of the epithelial cells of many organs. It is responsible for the integrity of the mucosal tissue, the first line of defence against environmental toxic molecules [102].

The study by Chowdhury et al. [103] confirmed the role of β-catenin in differentiating the respective grades of dysplasia. The concentration of β-catenin increased in the individual grades of dysplasia. In the study by Prgomet et al. [104], there were statistically significantly higher expressions of β-catenin in dysplasia compared with normal-appearing oral mucosa. Still, the authors did not compare the results in different grades of oral dysplasia. Decreased E-cadherin and increased VEGF expression could be involved in the tissue growth and transformation of OPMDs, correlating with their different histological grades in numerous studies. This was confirmed in the study by Sharada et al. [105], as these association markers can be used to predict the potential risk of malignant transformation in OED. In their research, Sharma et al. [106] also evaluated the importance of E-cadherin in differentiating the dysplasia grade. E-cadherin expression decreased significantly with increasing dysplasia grade.

Similarly, Puneeta et al. [107] assessed the expression of vimentin and E-cadherin in different grades of OED and OSCC. In the OED group, a progressive involvement of all layers was observed, with 5% of mild OED, 10% of moderate OED and 70% of severe OED showing expression of E-cadherin up to the superficial layers, which was statistically significant. Vimentin expression was low in mild OED, while high expression was more prevalent in moderate and severe OED. This finding was statistically significant. Furthermore, the study by Miguel et al. [108] aimed to investigate the immunoexpression of matrix metalloproteinase 9, tissue inhibitor of metalloproteinase 1, and vimentin. The authors confirmed the role of the epithelial expression of vimentin in the malignant process. Also, they found that smokers had a higher epithelial expression of MMP-9 and vimentin.

As mentioned earlier, N-cadherin is upregulated while E-cadherin is downregulated during EMT in carcinogenesis. This process is associated with enhanced migratory and invasive traits, which causes an inferior patient survival rate [90]. Chandolia et al. [109] assessed N-cadherin expression in 100 cases (epithelium with normal oral mucosa, OED lesions, and OSCC). The differences were statistically significant, and the study showed that N-cadherin expression was more evident than in OED, followed by the normal oral epithelium.

Importantly, the Wnt pathway stabilises the ß-catenin protein and interferes in the ß-catenin and E-cadherin complex. The Wnt pathway is involved in the dysplastic changes that downregulate E-cadherin by TWIST (Figure 2). TWIST binds to E-cadherin and suppresses the transcription of E-cadherin [110]. Qahtani et al. [111] examined the expression of the TWIST protein. The authors found significant differences between severe dysplasia and other grades of oral dysplasia. The study confirmed that the cadherin–catenin complex and the proteins involved in their regulation play a role in carcinogenesis.

Podoplanin (PDPN) is a small cell-surface mucin-like glycoprotein [112]. Podoplanin expression is upregulated in different cell types, including fibroblasts, macrophages, T helper cells, and epithelial cells, during inflammation and cancer, where it plays important roles [113]. Podoplanin interacts with other proteins in the same or neighbouring cells. The binding of podoplanin to ligands leads to the modulation of signalling pathways, which regulate proliferation, contractility, migration, epithelial–mesenchymal transition, and the remodelling of the extracellular matrix [114]. Karunagaran et al. [115] showed a significant association between dysplasia and podoplanin expression, with increasing dysplasia grade corresponding with podoplanin expression. Podoplanin seemed to have an increased expression as the dysplasia grade increased, suggesting its role in the progression of the disease toward malignancy. Lunawat et al. [116] investigated podoplanin immunoexpression in lymphatic vessels of OED. Podoplanin expression significantly increased with higher grades of dysplasia. This observation might help to diagnose the wider progression of dysplastic lesions to carcinoma. The study by Monteiro et al. [117] aimed to evaluate the expression of biomarkers CD44v6, CD147, EGFR, p53, p63, p73, p16, and podoplanin in oral leukoplakia. In a multivariate analysis, the authors observed a significant increase in high expression from normal tissue to low-grade dysplasia and high-grade dysplasia cases in CD44v6, p53, p73, and podoplanin. In conclusion, podoplanin expression could be a useful predictive marker in malignant transformation. Similarly, Abidullah et al. [118] found that the staining of MUC4 increased from mild to moderate to severe dysplasia. Mucin MUC4 is membrane-associated and plays a protective role [119]. Therefore, MUC4 can be a marker for the diagnosis of OED.

### 2.3. Biomarkers Related to Cell Death Regulation

Other altered proteins are the members of the Bcl-2 family. These proteins are considered as the principal players in the cascade of events that activate or inhibit apoptosis [120]. In this family, there are, for example, Bcl-XL, Bcl-2, and Bax. Bcl-2 acts as a checkpoint upstream of caspases and mitochondrial dysfunction [121]. Also, Bcl-2 can rescue maturation at several points of lymphocyte development. The Bcl-2 proto-oncogene was discovered at the chromosomal breakpoint of t (14;18) found in a human follicular lymphoma [122]. Pathak et al. [123] observed that the level of Bcl-2 increased with the grade of dysplasia. However, Bcl-2 expression was decreased in OSCC. Pallavi et al. [124] assessed the expression of Bcl-2 and c-Myc in OED and OSCC. Similarly, the authors noticed that Bcl-2 increased with grades of dysplasia. Bcl-2 proteins could positively affect lesion progression from premalignancy to malignancy.

Also, the PD-1/PD-L1 pathway can be a potential marker for oral dysplasia. Programmed Cell Death Protein 1 (PD-1) inhibits immune responses and modulates T-cell activity [125]. Kujan et al. [126] investigated the role of the PD-1/PD-L1 pathway in the development of dysplasia and OSCC. The study found that the PD-1/PD-L1 pathway can be associated with the development of OSCC and the grade of dysplasia. Programmed cell death 4 (PDCD4) functions as a tumour suppressor and an inhibitor of protein translation [127]. PDCD4 expression was observed in normal oral mucosa, OED, and OSCC. Desai and Kale [128] showed that the maximum expression was observed in normal oral mucosa, which reduced significantly in OED and OSCC. 

Heat shock protein 27 (HSP27) belongs to the small-molecular-weight heat shock protein family and has a molecular weight of approximately 27 KDa [129]. This protein protects other proteins from damage due to environmental factors such as heat, toxins, free radicals, and ischaemia [130]. Karri et al. [131] found that a low expression of HSP27 could be an early molecular indicator of initial dysplastic changes in normal mucosa. Conversely, the overexpression of HSP27 could be a prognostic value of malignant transformation from oral dysplasia to oral squamous cell carcinoma. Cornulin (known as C1 Orf10, or squamous epithelial heat shock protein 53) is a member of the heat shock protein 70 (HSP70) family [132]. Cornulin plays an important role in the differentiation of the epidermis. The expression of cornulin causes cell cycle arrest at G1, and its downregulation plays a role in oral carcinogenesis [133]. Santosh et al. [134] found that cornulin expression decreased in oral dysplasia compared with normal oral mucosa and was absent in OSCC.

### 2.4. Biomarkers Related to Cellular Metabolism

A major component of the cellular response to oxygen deprivation is the transcription factor HIF-1 (hypoxia-inducible factor-1). HIF-1 consists of an HIF-1 beta unit and one of three units of HIF-1alpha, HIF-2alpha, or HIF-3alpha [135]. Patel et al. [136] assessed the expression of HIF-1alpha in OED and compared the expression between grades. The authors noticed that the expression of HIF-alpha statistically significantly increased as grades of oral dysplasia were higher. Also, HIF-alpha could be a marker of risk of malignant transformation.

Inducible nitric oxide synthase (iNOS) is an enzyme in oxygen and nitrogen metabolite metabolism [137]. Using immunohistochemical methods, Singh et al. [138] compared iNOS expression between oral leukoplakia and OSCC. The authors found that the expression of iNOS rose with the progressing clinical stages of oral leukoplakia and OSCC. Therefore, iNOS might be a diagnostic marker in oral leukoplakia and a prognostication marker of OSCC. Another enzyme, cyclooxygenase (COX or prostaglandin–endoperoxide synthase), is required to change arachidonic acid to prostaglandins [139]. Sharada et al. [140] examined the expression of COX-2 and type IV collagen in OED. The study found that its expression increased significantly as the grade of dysplasia was higher. This marker could be applied to assess the malignant potential.

### 2.5. Biomarkers Related to Extracellular Signalling Pathways

Paxillin is a 68 kDa, phosphotyrosine-containing protein that may play a role in several signalling pathways [141]. The study by Alam et al. [142] presented a statistically significant correlation between increased grades of oral dysplasia and expression of paxillin. Paxillin may play an important role in the pathogenesis of oral dysplasia and OSCC. 

EGFR is a 170 kDa transmembrane glycoprotein receptor [143]. EGFR regulates cell growth, differentiation, and gene expression [144]. Fakurnejad et al. [145] demonstrated that an anti-EGFR agent could successfully discriminate high-grade dysplastic lesions from low-grade dysplasia. Melanoma inhibitory activity (MIA) and MIA2 are other receptors participating in tumour growth and invasion. Kawai et al. [146] evaluated MIA and MIA2 as expressed in the oral mucosa within early neoplastic lesions and suggested that MIA and MIA2 are useful novel immunohistochemical markers for discriminating between normal tissue and OED.

Laminins are another family of structural proteins. Laminins participate in organising the complex interactions of the basement membranes. Laminin-1 is in the Reichert membrane (extraembryonic basement membrane) [147]. A study by Vageli et al. [148] assessed laminin immunostaining in biopsies as a useful biomarker of actinic cheilitis and differential diagnosis between actinic cheilitis and lip cancer. This marker can differentiate between low- and high-grade dysplasia. This study can provide new insight into the mechanism of progression of actinic cheilitis into lip cancer. Also, Nguyen et al. [149] evaluated the immunoexpression of LAMC2. The expression of LAMC2 was significantly associated with the grade of dysplasia. LAMC2 may be a predictive marker for the malignant progression of leukoplakia.

In the study by Debta et al. [150], GLUT-1 also appeared as a marker for differentiating dysplasia severity. A statistically significant increasing level of GLUT-1 corresponded to more advanced grades of dysplasia and was consistent with the WHO system. GLUT-1 expression was significantly increased from normal to mild, moderate, and severe dysplasia. The expression of the GLUT-1 marker complemented the WHO grading system of OED. Also, Patlolla et al. [151] confirmed a significant correlation between the location of GLUT-1 within the cell and the grade of dysplasia.

Moreover, Udompatanakorn and Taebunpakul [152] assessed the pattern of expression of METTL3 in OED. METTL3 is an enzyme involved in the post-transcriptional methylation of internal adenosine residues [153]. The authors observed that the expression of METTL3 increased in oral dysplasia and OSCC. METTL3 expression might be a marker for the progression of oral dysplasia and transformation to OSCC.

Another marker is the minichromosome maintenance protein (MCM-2), which is a key component of the pre-replication complex. This protein may be involved in the formation of replication forks and in the migration of other proteins during DNA replication [154]. The study by Zakaria et al. [155] aimed to assess MCM-2 activity in oral epithelial dysplastic lesions. The MCM-2 immunostaining showed a statistically significant increase from mild to severe dysplasia, and the highest value was in invasive squamous cell carcinoma. MCM-2 activity is associated with the grade of dysplasia. This observation suggests that MCM-2 may be a potential biomarker for early squamous cell carcinoma.

### 2.6. Limitations and Challenges

The limitations of this review include the heterogeneity of the study designs in terms of clinical and histopathologic diagnoses, as well as laboratory methods determining markers of oral dysplasia. The included studies focused on a wide range of phenomena detected using immunochemical methods. However, changes in EMT markers (i.e., cadherin, vimentin, etc.) and p53 or Ki-67 were most frequently described. These molecules are known to affect the cell cycle, proliferation, and differentiation of cells, including cancer cells. Laboratory testing is important in assessing the levels of markers, but the results may be influenced by the quality of the collected specimens and the storage time.

It should be noted that the pathologists manually assigned the different degrees of dysplasia. The quality of the collected samples, the experience of the researchers, and the type of classification can impact the findings. The complexity of cell tumorigenesis and the number of pathways involved in this process (considering the relationships between different pathways) creates a problem in identifying a single universal marker for OED grading. Therefore, developing immunohistochemical marker panels with high sensitivity and specificity to detect early stages of oral dysplasia should be considered in the future.

To summarise this review, we include Table 2, presenting the main potential immunohistochemical markers for oral dysplasia. In the Supplementary Materials, we attach Table S1, reporting all potential immunohistochemical markers with methodological descriptions of the tested samples.

## 3. Conclusions

According to our review, there are many various immunohistochemical biomarkers for dysplasia grading. The researchers most commonly used p53 protein, Ki-67 protein, cadherins/catenins, and other proteins as markers to differentiate grades of oral epithelial dysplasia. However, further research is desirable to confirm these outcomes and detect new potential biomarkers to properly establish the dysplasia grade and the risk of malignant transformation in a minimally invasive way.

## Figures and Tables

**Table 1:** The WHO diagnostic criteria for oral epithelial dysplasia—update 2022—according to Muller and Tilakaratne [10].

Architectural Features	Cytological Features
Irregular epithelial stratification	Abnormal variation in nuclear size
Loss of polarity of basal cells	Abnormal variation in nuclear shape
Drop-shaped rete ridges	Abnormal variation in cell size
Mitoses high in epithelium	Abnormal variation in cell shape
Generalised premature keratinisation	Increased N/C ratio
Keratin pearls within rete ridges	Atypical mitotic figures
Loss of epithelial cell cohesion	Increased number and size of nucleoli
Altered keratin pattern for oral sub-site	Hyperchromasia
Verrucous or papillary architecture	Increased number of mitotic figures
Extension of changes along minor gland ducts	Single-cell keratinisation
Sharply defined margin of changes	Apoptotic mitoses
Multiple different patterns of dysplasia	Increased nuclear size
Multifocal or skip lesions	
Expanded proliferative compartment	
Basal cell clustering/nesting	

**Table 2:** Summary of main potential immunohistochemical biomarkers for assessing grading of oral dysplasia.

Relation of Biomarkers	Examples of Biomarkers for Grading of Oral Dysplasia
Cell division and proliferation	p53 [53,54,55,75,76,117], Ki-67 [56,68,69,70,71,72,73,74,75,76], CD105 [73,74], p63 [39,42,117], CD31 [39], CD34 [43], cycD1 [42], VEGF [43], YAP, Np63 [59], stathmin [80], CDKN1A [53]
Epithelial–mesenchymal transition	CK5\6 [96], CK19 [98], β-catenin [103,104], N-cadherin [109], E-cadherin [104,105,106,107], TWIST [111], VIM [107,108], PDPN [115,117], MMP-9 [108]
Cell death regulation	Bcl-2 [123,124], PDCD4 [128], HSP27 [131], cornulin [134]
Cellular metabolism	HIF-1a [136], iNOS [138], COX-2 [140]
Extracellular signalling pathways	Paxillin [142], EGFR [145], MIA, MIA2 [146], laminin [148], LAMC2 [149], GLUT-1 [150,151], METTL3 [152], MCM2 [155], 8-OHdG, Ref-1, XRCC-1 [156], Orai1, STIM1 [32], NANOG [30,31]

Legend: 8-OHdG, 8-hydroxy-2-deoxyguanosine; Bcl-2, B-cell lymphoma 2; CD, cluster differentiation; CDKN1A, cyclin-dependent kinase inhibitor 1A; CK, cytokeratin; COX-2, cyclooxygenase 2; CycD1, cyclin D1; EGFR, epidermal growth factor receptor; GLUT-1, glucose transporter-1; HIF, hypoxia-inducible factor; HSP, heat shock protein; iNOS, inducible nitric oxide synthase; LAMC2, laminin subunit gamma 2; MCM2, minichromosome maintenance complex component 2; METTL3, methyltransferase-like 3; MMP-9, matrix metalloproteinase 9; MIA, melanoma inhibitory activity; PDCD4, programmed cell death 4; PDPN, podoplanin; Ref-1, Redox factor-1; STIM1, stromal interaction molecule 1; VEGF, vascular endothelial growth factor; VIM, vimentin; XRCC-1, X-ray Repair Cross Complementing-1; YAP, Yes-associated protein.

## Supplementary Materials

The following supporting information can be downloaded at https://www.mdpi.com/article/10.3390/biomedicines12030577/s1: Table S1. Detailed summary of potential immunohistochemical biomarkers for assessing grading of oral dysplasia with a brief description of the sample characteristics and the study setting.
