# Supplementary material for: Potential Immunohistochemical Biomarkers for Grading Oral Dysplasia: A Literature Review
Source: Biomedicines. 2024 Mar 5;12(3):577. doi: 10.3390/biomedicines12030577 (PMC10967812; doi:10.3390/biomedicines12030577)
Supplement: Supplementary file 1 [file biomedicines-12-00577-s001.zip › biomedicines-2900699-supplementary.pdf]

Table S1. Detailed summary of potential immunohistochemical biomarkers for assessing grading of oral dysplasia with a brief description of the sample characteristics and the study setting.

Study	Setting	Sample characteristics	Potential biomarker
Abidullah et al., 2019	India	OED (n=45): mild (n=15), moderate (n=15), severe (n=15); normal (n=10)	MUC4
Alam et al., 2022	India	OD (n=20): mild (n=10), moderate (n=8), severe (n=2); OPMD (n=20); OSCC (n=20); normal (n=20)	paxillin
Aravind et al. 2017	India	OD (n=10): mild (n=3), moderate (n=3), severe (n=4); OSCC (n=30); hyperplasia (n=10); normal (n=10)	OPN
Barros et al., 2022	Brazil	OED: mild (n=14), moderate (n=10), severe (n=3), without dysplasia (n=17)	8-OHdG, Ref-1, XRCC-1
Batool et al., 2020	Pakistan	OD (n=60): mild (n=20), moderate (n=21), severe (n=19); normal (n=60)	CK5\6
Bavle et al., 2021	India	OD (n=19): mild (n=15), moderate (n=3), severe (n=1); simple hyperplasia (n=2)	p63, CD31
Chandolia et al., 2017	India	OED (n=60): mild (n=20), moderate (n=20), severe (n=20); OSCC (n=30); normal (n=10)	N-cadherin
Chowdhury et al., 2021	India	OED (n=20): mild (n=10), moderate (n=5), severe (n=5); OSMF (n=10); normal (n=10)	β-catenin
Dash et al., 2020	India	OED (n=45): mild (n=15), moderate (n=15), severe (n=15); OSCC (n=45); normal (n=15)	Ki-67
de Vicente et al., 2019	Spain	OED (n=55): low grade (n=42), high grade (n=13); OSCC (n=125)	NANOG
Debta et al., 2020	India	OSMF (n=7): no dysplasia (n=3), mild (n=2), moderate (n=2); OL (n=11): no dysplasia (n=4), mild (n=5), moderate (n=2); erythroplakia (n=2): moderate (n=2); OLP (n=7): no dysplasia (n=4), mild (n=2), moderate (n=1); normal (n=10)	GLUT-1
Desai et al., 2017	India	OED (n=60): mild (n=20), moderate (n=20), severe (n=20); OSCC (n=30); normal (n=10)	PDCD4
Fakurnejad et al., 2019	USA	OD (n=101): low-grade (n=53), high-grade (n=48); malignant regions (n=50); normal (n=68)	EGFR
Gadbail et al., 2018	India	OSMF (n=50): low-risk (n=28), high-risk (n=22); normal (n=30)	Ki-67, CD105, α-SMA antibodies
Gadbail et al., 2017	India	OPMD (n=140): OED (n=115): mild (n=44), moderate (n=43), severe (n=28); hyperkeratosis/atrophic (n=25); normal (n=30)	Ki-67, CD105, α-SMA antigen
Grubelnik et al., 2020	Slovenia	OD: low-grade (n=36), high-grade (n=36); OSCC (n=30); normal (n=15)	NANOG
Gupta et al., 2017	India	OED (n=30): mild (n=10), moderate (n=10), severe (n=10); normal (n=10)	VEGF, CD34
Imaizumi et al., 2021	Japan	OED: mild (n=62), moderate (n=23), severe (n=12); OSCC (n=61)	53BP1, Ki-67
Kamala et al., 2022	India	OSMF (n=35): no dysplasia (n=14), OED (n=21): mild (n=4), moderate (n=4), severe (n=13); OSCC (n=10); normal (n=10)	Ki-67
Karri et al., 2020	India	OED (n=30): mild (n=10), moderate (n=10), severe (n=10); OSCC (n=10); normal (n=10)	HSP27
Karunagaran et. al., 2019	India	OSMF (n=30): mild (n=10), moderate (n=10), severe (n=10); normal (n=10)	PDPN
Kawai et al., 2021	Japan;	OED (n=60): low-grade (n=30), high-grade (n=30); OSCC (n=30); normal (n = 10)	MIA, MIA2

Kujan et al., 2022	Australia	OED (n=53): low-grade (n=24), high-grade (n=29); OSCC (n=14); non-dysplastic (n=34)	FOXP3, TGF-beta, IL-6, IL-10
Leung et al., 2017	United Kingdom	OED (n=73): mild (n=24), moderate (n=17), severe (n=32); OSCC (n=13); normal (n=24)	Ki-67, CD1, H2AX, p53 and p16, H3K9me3
Lunawat et al., 2021	India	OED (n=30): mild (n=15), moderate (n=9), severe (n=6); normal (n=30)	PDPN
Miguel et al., 2021	Brazil	OED (n=66): low-risk (n=42), high-risk (n=24); OSCC (n=27); non-neoplastic (n=28)	MMP-9, TIMP-1, VIM
Mondal et al., 2020	India	OL (n=140): OED (n=126): mild (n=64), moderate (n=43), severe (n=29); definite malignancies (n=14)	Ki-67
Monteiro et al., 2021	Portugal	OL-OED (n=52): low-grade (n=41), high-grade (n=11); normal (n=12)	BubR1, Mad2, Bub3, Spindly
Monteiro et al., 2022	Portugal	OL-OED (n=52): low-grade (n=41), high-grade (n=11); normal (n=12)	CD44v6, CD147, EGFR, p53, p63, p73, p16, PDPN
Nguyen et al. 2017	Japan	OL-OED (n=93): mild (n=11), moderate (n=78), severe (n=4); OSCC (n=99)	LAMC2
Ono et al., 2019	Japan	OED (n=100): low-grade (n=50), high-grade (n=50); CIS (n=50); OSCC (n=80); hyperplasia (n=20); normal (n=20)	YAP, Np63
Pallavi et al., 2018	India	OED (n=30): mild (n=10), moderate (n=10), severe (n=10); OSCC (n=30); normal (n=10)	Bcl-2, c-Myc
Pandya et al., 2018	India	OED (n=24); OSCC (n=28)	p53, CDKN1A
Patel et al., 2017	India	OL-OED (n=30): mild (n=8), moderate (n=9), severe (n=13); OSCC (n=30)	cycD1, p63
Patel et al., 2019	India	OED (n=30): mild (n=13), moderate (n=11), severe (n=6); OSCC (n=30)	HIF-1a
Pathak et al., 2022	India	OED (n=24): mild (n=8), moderate (n=8), severe (n=8); OSCC (n=24)	Bcl-2
Patil et al., 2022	India	OED (n=30): mild (n=10), moderate (n=10), severe (n=10); normal (n=10)	p53
Patlolla et al., 2020	India	OED (n=30): low-risk (n=15), high-risk (n=15); OSCC (n=45); normal (n=15)	GLUT-1
Prgomet et al., 2017	Sweden	OD (n=21): mild (n=5), moderate (n=8), severe (n=8)	WNT5A, E-cadherin, β-catenin
Puneeta et al., 2022	India	OED (n=60): mild (n=20), moderate (n=20), severe (n=20); OSCC (n=60); normal (n=5)	E-cadherin, VIM
Qahtani et al., 2020	Saudi Arabia	OED (n=15): mild (n=6), moderate (n=4), severe (n=5); OSCC (n=20)	TWIST
Rajeswari et al., 2020	India	OED (n=30): mild (n=10), moderate (n=10), severe (n=10); OSCC (n=10); hyperplasia (n=10); normal (n=10)	CK19
Santosh et al., 2019	USA	OED (n=50): low-grade (n=25), high-grade (n=25); OSCC (n=25); normal (n=25)	cornulin
Sawada et al., 2022	Japan	OED (n=40): mild (n=21), moderate (n=13), severe (n=6)	p53
Sharada et al., 2021	India	OED (n=30): mild (n=10), moderate (n=10), severe (n=10); OSMF (n=10); OSCC (n=10); normal (n=10)	COX-2
Sharada et al., 2018	India	OED (n=30): mild (n=10), moderate (n=10), severe (n=10); OSMF (n=10); OSCC (n=10); normal (n=10)	E-cadherin, VEGF
Sharma et al., 2022	India	OED (n=20); OSCC (n=20); normal (n=20)	E-cadherin
Singh et al., 2020	India	OL-OED (n=30): mild (n=9), moderate (n=10), severe (n=11); OSCC (n=30); normal (n=30)	iNOS
Suwasini et al., 2018	India	OL-OED (n=15): mild (n=5), moderate (n=5), severe (n=5); normal (n=5)	p53, Ki-67
Swain et al., 2022	India	OED (n=30): mild (n=10), moderate (n=10), severe (n=10); OSCC (n=30); normal (n=10)	Ki-67, MCM2

Takkem et al., 2018	Syria	OED (n=30): mild (n=10), moderate (n=10), severe (n=10); OSCC (n=30); normal (n=10)	Ki-67
Udompatanakorn&Taebunpakul, 2022	Thailand	OED (n=20): low-grade (n=10), high-grade (n=10); OSCC (n=20); normal (n=20)	METTL3
Vadla et al., 2022	India	OL-OED (n=30): mild (n=16), moderate (n=10), severe (n=4); OSCC (n=30); normal (n=30)	stathmin
Vageli et al., 2022	Greece	actinic cheilitis (n=34): low-grade ED (n=31), high-grade ED (n=3); LSCC (n=12)	laminin
Venkat Naga et al., 2019	India	OED (n=30): mild (n=10), moderate (n=10), severe (n=10); normal (n=10)	CD44 antibody
Wang et al., 2022	Taiwan	OPMD-OED (n=33): mild (n=19), moderate-severe (n=14); OSCC (n=66); normal (n=14)	Orai1, STIM1
Zakaria et al., 2018	Egypt	OED (n=30): mild (n=10), moderate (n=10), severe (n=10); early invasive OSCC (n=5); hyperplasia (n=5)	MCM2

Legend: 53BP1, P53-binding protein 1; 8-OHdG, 8-hydroxy-2-deoxyguanosine; α-SMA, α-smooth muscle actin; Bcl-2, B-cell lymphoma 2; BUBR1, budding uninhibited by benzimidazole-related 1; CD, cluster differentiation; CDKN1A, cyclin dependent kinase inhibitor 1A; CK, cytokeratin; COX-2, cyclooxygenase 2; CycD1, cyclin D1; ED, epithelial dysplasia; EGFR, epidermal growth factor receptor; FOXP3, forkhead box P3; GLUT-1, glucose transporter-1; H2AX, H2A histone family member X; H3K9me3, trimethylhHistone H3 (Lys9); HIF, hypoxia-inducible factor; HSP, heat shock protein; IL, interleukin; iNOS, inducible nitric oxide synthase; LAMC2, laminin subunit gamma 2; LSCC, lip squamous cell carcinoma; Mad2, mitotic arrest deficient 2; MCM2, minichromosome maintenance complex component 2; METTL3, methyltransferase-like 3; MMP-9, matrix metalloproteinase 9; MIA, melanoma inhibitory activity; MUC, mucin; OD, oral dysplasia; OED, oral epithelial dysplasia; OL, oral leukoplakia; OLP, oral lichen planus; OPMD, oral potentially malignant disorder; OPN, osteopontin; OSCC, oral squamous cell carcinoma; OSMF, oral submucous fibrosis; PDCD4, programmed cell death 4; PDPN, podoplanin; Ref-1, Redox factor-1; STIM1, stromal interaction molecule 1; TGF, transforming growth factor; TIMP-1, tissue inhibitor of metalloproteinase-1; VEGF, vascular endothelial growth factor; VIM, vimentin; XRCC-1, X-ray Repair Cross Complementing-1; YAP, Yes-associated protein
